# Precision design of dextran-permeated agarose hydrogels matching adipose stem cell adhesion timescales

**DOI:** 10.1016/j.mtbio.2025.101832

**Published:** 2025-05-06

**Authors:** Nicole Guazzelli, Ludovica Cacopardo, Arti Ahluwalia

**Affiliations:** a Research Center ‘E. Piaggio’, University of Pisa, Italy; b Department of Information Engineering, University of Pisa, Italy; c Centro 3R, Italy

## Abstract

Viscoelasticity is now recognised as a key parameter in modulating cell behaviour. Tailoring time-dependent materials to elicit specific cellular responses is, however, a challenge because of the intricate relationship between the substrate relaxation time (τ_rel_) and the cell sensing time-window which depends on the time required for the formation of focal adhesions (τ_b_) and the duration of their lifetime (τ_L_). Here, we introduce a novel design approach to guide cell behaviour based on the cell-perceived Deborah number, De = τ_rel_/τ_L,_ arguing that for De > 1 and De < 1, substrates promote cell differentiation because stable adhesions and sustained tension drive mechanotransduction and lineage-specific differentiation on the basis of substrate stiffness. Instead, cell stemness is maintained in the De ∼1, whereby excessive mechanical signalling is prevented as cells balance adhesion stability and plasticity. The design workflow consists in modelling substrate τ_rel_, enabling the selection of the optimal gel formulation according to the cell-perceived De.

The workflow was applied to agarose gels with different dextran concentrations in the liquid phase, which act as modulators of mechanical time-dependent properties. To predict the relaxation times for these gels, we developed an in-silico model which integrates their structural and transport properties. Our results show that the gels have an almost constant equilibrium elastic modulus, while their τ_rel_ decreases with increasing dextran concentration in the liquid phase.

Considering adipose-derived mesenchymal stem cells (ADSCs) and their characteristics sensing times, we defined dextran concentrations to mimic the different De conditions in the agarose gels. Experimental cell investigations confirmed the validity of the design approach: ADSC differentiation, highlighted by YAP nuclear translocation, was promoted in the case of De < 1 and De > 1, respectively eliciting adipogenic and osteogenic lineages. On the other hand, cells maintained their stemness when De ∼1.

This study provides novel insights on the interplay between hydrogel viscoelasticity and cellular behaviour and paves the way for precision design of viscoelastic biomaterials for in-vitro studies and regenerative medicine.

## Graphical abstract

**Figure:**
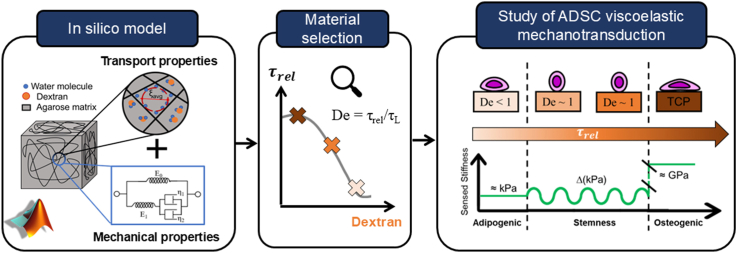

## 1 Introduction

That cells respond to the viscoelasticity of substrates is now well known. Recent studies have demonstrated that cell proliferation, migration and differentiation are influenced by the stress relaxation rate of soft substrates. Hydrogels with faster stress relaxation rates enhance cell spreading and proliferation, while slower stress relaxation restricts cell movement and proliferation [[1], [2], [3]]. Moreover, it has been observed that as matrix dissipative properties increase, the probability of hepatic stellate cell differentiation decreases [4]. Hydrogels with tunable viscoelastic properties have also been used to guide neural stem cell differentiation, demonstrating that faster stress relaxation promotes neuronal fate, whereas slower relaxing matrices favour glial differentiation. In a different study, mesenchymal stem cells cultured on viscoelastic hydrogels showed enhanced osteogenic differentiation on substrates with slower stress relaxation, mimicking the mechanical environment of bone tissue [[5], [6], [7]]. Moreover, hydrogels with controlled viscoelastic properties regulate stem cell lineage commitment in 3D cultures [8,9]. However, despite the wealth of recent studies investigating “viscoelastic mechanotransduction” [10], we lack a strategy for precision design of viscoelastic substrates for directing cell differentiation or stemness [3,[11], [12], [13], [14], [15]].

Cell mechanosensing and transduction is largely attributed to focal adhesions (FAs): protein complexes linking the cytoskeleton to adhesion ligands in the extra-cellular matrix [[16], [17], [18]]. The FA binding time constant (τ_b_), which is typically fixed for each cell type, represents the characteristic time required for FA formation and is inversely related to the rate of focal adhesion binding to the matrix. On the other hand, FA lifetime (τ_L_) represents the time for which FAs remain intact and functional before disassembly or reorganization. τ_L_ can be modulated by environmental conditions [11,[19], [20], [21], [22], [23], [24]]: it increases when the cells sense substrate forces that are stable over time, while it decreases in the case of unstable time-varying forces. The range of the cell sensing time-window or the cell observation time (i.e. the time that passes from its binding to the substrate and its unbinding) thus depends on τ_L._

**Fig. 1:**
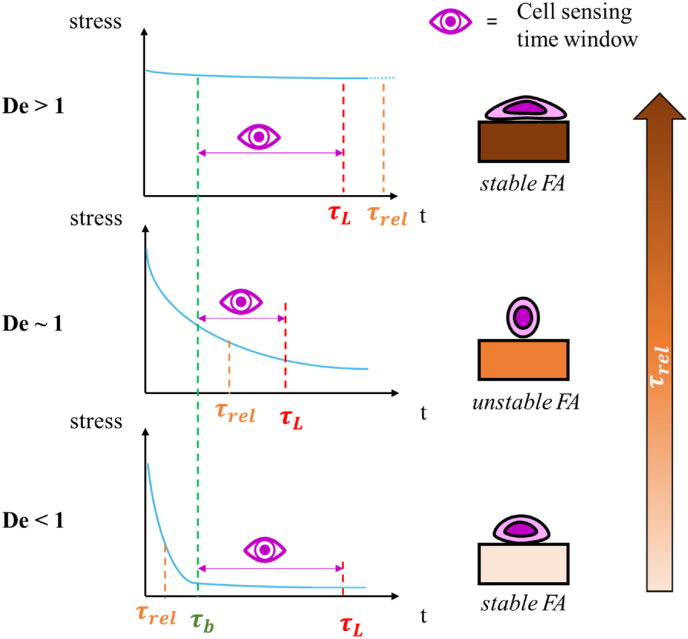
Schematic of the relationship between cell sensing times and substrate relaxation times: three different conditions can be defined according to the cell-perceived Deborah number (De = τ_rel_/τ_L_). If De is less than or greater than 1, then the cell perceives a substrate with a constant stress, while if De is close to 1, the cell perceives a substrate with a time varying state of stress.

The relaxation time of a substrate (τ_rel_) also has a key role in cell mechanosensing because it affects stress transmission to the cytoskeleton through the FAs. In particular, it determines how fast the stress in the substrate decreases from its instantaneous value (higher stress) to its equilibrium response (lower stress) [[1], [2], [3]]. To understand how cells interpret substrate viscoelasticity and therefore design gels able to elicit a specific cellular response, it is crucial to consider the relationship between their sensing time-window and τ_rel_. We thus defined the cell-perceived Deborah number as De = τ_rel_/τ_L_, identifying three main conditions (Fig. 1):

i) De > 1, occurring when τ_rel_ is longer than τ_L_ (τ_rel_ > τ_L_): substrate relaxation is so slow that the mechanical traction forces on the cells remains almost constant. This stable mechanical environment promotes stress fibre formation and consolidation, reinforcing cell adhesion. It is typically found in elastic substrates such as tissue culture plastic (TCP).
ii) De < 1, when τ_rel_ is shorter than τ_L_ (τ_rel_ < τ_L_): the substrate rapidly relaxes from the instantaneous to the equilibrium state. This means that cells sense a substrate that is stable over time because the viscoelastic dynamics are over before cell binding. Similarly to the previous case, the constant equilibrium stress, albeit lower in magnitude, stabilises FA promoting cell adhesion.
iii) De ∼1, referring to case in which τ_rel_ ∼ τ_L_ and cells perceive a substrate which changes over time destabilising FAs and hindering cell adhesion. Notably, this intermediate condition corresponds to the case in which FAs are less stable (shorter τ_L_), while both De < 1 and De > 1 are characteristic of longer τ_L._

The interplay between substrate relaxation time and cellular sensing time directly influences cell mechanotransduction through the transcriptional regulators YAP and TAZ. Specifically, YAP/TAZ translocate to the nucleus in response to increased actin tension on stiffer substrates, activating cellular pathways for proliferation, migration and differentiation [[25], [26], [27], [28]]. This highlights the relevance of defining substrate properties according to the cell-perceived De. To achieve this goal, we conceived a novel gel design approach based on a computational representation of its time-dependent properties, enabling the selection of specific substrate τ_rel_ to obtain the desired De condition.

**Fig. 2:**
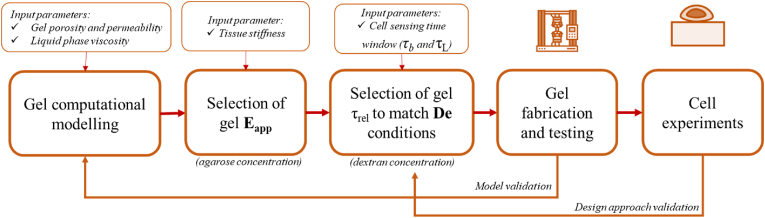
Workflow for the design of dextran-permeated agarose hydrogels with different cell-perceived De ranges. The starting hypothesis is that the cell-perceived De can modulate cell behaviour. Input parameters are first obtained from the literature or derived experimentally. The computational model, which predicts material viscoelastic behaviour, is used to select gels with appropriate De ranges. Gels with different De conditions are thus fabricated to probe cell responses to see if they concur with our hypothesis.

Although models for predicting the viscoelastic behaviour of gels according to their composition or structure have made significant strides, several limitations and critical points remain [10,[29], [30], [31]]. Many models, especially those based on energy balance or finite element methods [29,30,[32], [33], [34], [35]], can require substantial computational resources, limiting their applicability for large-scale simulations or real-time predictions. Some advanced computational models may require expertise in numerical methods, programming languages, or material science principles for their implementation and interpretation. Often, they rely on input parameters that may be challenging to measure experimentally or that have inherent variability. Sensitivity to these parameters can introduce uncertainty in model predictions and limit their reliability [30,[32], [33], [34],^36^,^37^]. As summarised in Fig. 2, our approach combines easily measurable structural and rheological material parameters (gel permeability and porosity and liquid phase viscosity) with cell adhesion characteristic times (τ_L_ and τ_b_) and tissue stiffness as input parameters to predict substrate relaxation time (τ_rel_) as well as the instantaneous and equilibrium elastic moduli (E_inst_ and E_eq_).

The design workflow was applied to dextran-permeated agarose hydrogels. In a previous study, we observed that the gel viscous properties depend on the dextran concentration, while their equilibrium elastic properties remain unchanged [31]. Specifically, when liquid phase viscosity is increased, the instantaneous elastic response (E_inst_) and relaxation time (τ_rel_) of the hydrogels decrease with increasing dextran concentration [31]. To explain this behaviour, we proposed a mechanism based on the competition between agarose and dextran in binding to water molecules: dextran's high affinity with water results in the dissociation of water molecules from the agarose network [31,[38], [39], [40], [41]]. Dextran-bound water molecules are thus free to flow through the network pores when the hydrogel is compressed, promoting a more pronounced liquid-like behaviour. We modelled this behaviour, validating its output by comparison with experimental data.

Based on well-established mechanical and bond lifetime data on adipose derived mesenchymal stem cell (ADSC), the model was used to select substrate compositions with an agarose concentration corresponding to adipose tissue stiffness (2–6 kPa [[42], [43], [44], [45], [46], [47]]) and different dextran concentrations to obtain relaxation times corresponding to different cell-perceived De [25,[48], [49], [50], [51]]. Experimental investigations were finally performed on cell cultures to corroborate our De-based substrate design approach.

## 2 Materials and methods

### 2.1 Modelling agarose hydrogel viscoelastic behaviour

**Fig. 3:**
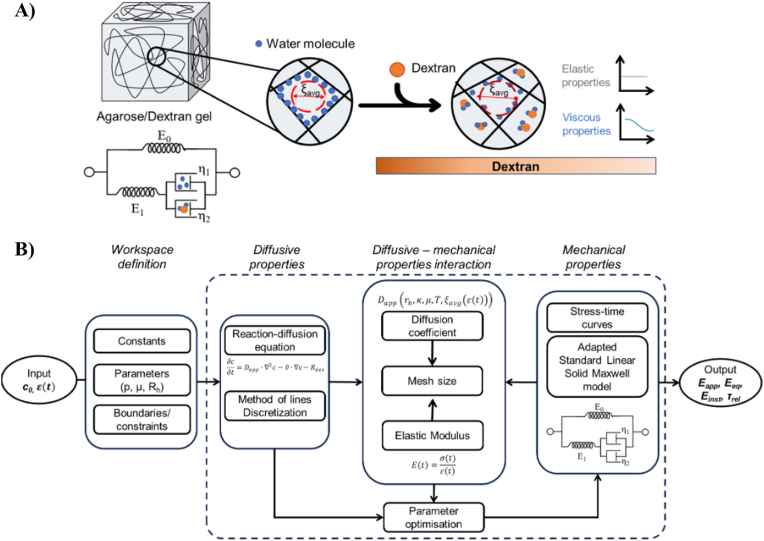
A) Schematic modelling of agarose/dextran gels; B) Outline of the in silico model developed for predicting ${E_{app},E}_{inst}$, $E_{eq}$, $\tau_{rel}$ of agarose hydrogels as a function of the initial dextran concentration in the liquid phase (c_0_) and the applied strain (ε(t)).

Our phenomenological model describes the transport of dextran through the agarose matrix, enabling the prediction of the hydrogel's apparent elastic modulus (E_app_) and of its viscoelastic descriptors (equilibrium and instantaneous elastic moduli and relaxation time, (E_eq_, E_inst_, and τ_rel_) as a function of variations in its liquid phase composition (Fig. 3). The in silico approach is structured in two steps: in the first we select the polymer concentration which gives a gel E_app_ corresponding to adipose tissue stiffness. The second step allows determining the dextran concentrations which generate gels matching the different De conditions on the basis of the sensing time window of ADSCs.

Agarose gels prepared under controlled conditions are known to be homogeneous [52]. The model describes the uniform agarose network by its average mesh size, i.e. the distance between two adjacent agarose crosslinks. The interaction between the molecular diffusion of dextran in the liquid phase and the structural and mechanical properties of the porous agarose network are correlated through the average mesh size, ${\;\xi }_{avg}\left(\varepsilon \left(t\right)\right)$ [53], which also puts a constraint on the maximum radius of molecules that can effectively diffuse through the pores of the system. ${\;\xi }_{avg}\left(\varepsilon \left(t\right)\right)$ depends on the degree of agarose gelation (α, which was set to 0.65 as reported in literature [38,[54], [55], [56], [57]]) and on the state of strain (*ε*(t)), which is time dependent because of the viscoelastic nature of the gel.

The mass transport of dextran in the liquid phase during a compression test was described through the reaction-diffusion equation (Eq. (1)), assuming that the agarose network is stable, the absence of convection, and that the reaction between water and agarose chains is at equilibrium. Here, the space and time dependent dextran concentration in the gel is represented by c and $R_{dex}$ is the reaction term. As a number of studies have shown that dextran does not bind to agarose in the absence of activators [[38], [39], [40], [41]], only the reaction between water and dextran molecules was considered; therefore, a zero-order condition $R_{dex}=k_{r}c_{0}$, was chosen ($c_{0}$ is the nominal concentration of dextran in the gel and $k_{r}$ is the dextran-water binding rate).

(1) $$\frac{\partial c}{\partial t}=D_{app}\cdot \nabla^{2}c-R_{dex}$$

We also assumed spherical dextran molecules [38,41] with a hydrodynamic radius r_h_ and an apparent diffusion coefficient $D_{app}$ (Eq. (2)) [58]. Its mathematical expression combines Brinkmann's theory of hindered diffusion in porous media [[56], [57], [58]], Carman-Kozeny equations for permeability [54,57,58], Flory's theory of rubber elasticity and the Amsden obstructing-scaling model [53,55,59]. The diffusion coefficient from the Stokes-Einstein equation - which depends on the solute viscosity (μ), $r_{h}$ and the temperature (T) – was thus integrated with additional factors accounting for the hindered permeability (κ) of the liquid phase through the pores of the agarose polymer network and the average mesh size, $\xi_{avg}\left(\varepsilon \left(t\right)\right)$ [53].

(2) $$D_{app}\left(r_{h},\kappa ,\mu ,T,\xi_{avg}\left(\varepsilon \left(t\right)\right)\right)=\frac{k_{b}Te^{-\left(\frac{r_{h}}{2\xi_{avg}\left(\varepsilon \left(t\right)\right)}\right)}}{6\pi \mu \;r_{h}\left[1+\frac{r_{h}}{\sqrt{\kappa }}+\frac{r_{h}^{2}}{3\kappa }\right]}$$

The time dependent mechanical behaviour of the hydrogel was then described through lumped parameter models implemented in Simulink 10.5 (see SI1 for more details). Since dextran modulates the liquid phase viscosity μ, we adapted the generalised Maxwell or standard linear solid model (SLS) [10] by including, a second dashpot (η_2_) in parallel with η_1_, modulated by a factor that accounts for the volumetric fraction of dextran (φ). The inset in Fig. 2 shows the adapted model (aSLS), while Eq. (3) reports its transfer function E(t) representing the time dependent elastic modulus as the ratio of the output stress response of material σ(t) and of an input strain ε(t), expressed as a constant strain rate input ($\dot{\varepsilon }$), according to the epsilon dot method [10,31].

(3) $$E\left(t\right)=\frac{\sigma \left(t\right)}{\varepsilon \left(t\right)}=\frac{\sigma \left(t\right)}{\dot{\varepsilon \;}t}=E_{0}+\frac{\left(\eta_{1}+{\varphi \cdot \eta }_{2}\right)}{t}\left({1-e}^{-\frac{E_{1}}{\left(\eta_{1}+{\varphi \cdot \eta }_{2}\right)}t}\right)$$

Finally, Eq. (4) reports the expression for the average mesh size $\xi_{avg}$ which is used to relate the state of strain ε(t) in Eq. (3) with the diffusive properties via Eq. (2).

(4) $$\xi_{\text{avg}}\left(t\right)={\left(\frac{18k_{B}T}{\pi \alpha^{\frac{2}{3}}E\left(t\right)}\right)}^{\frac{1}{3}}$$

The lumped parameters $E_{0},E_{1},\eta_{1},\eta_{2}$ were then combined as reported in Table 1 to derive the viscoelastic descriptors. The apparent elastic modulus (E_app_) was also computed as the slope of the stress-strain curves within the linear viscoelastic region (5 % strain), to select agarose concentration matching with adipose stiffness values reported in the literature [[42], [43], [44], [45], [46], [47]].

**Table 1:** Viscoelastic descriptors and lumped parameters of the aSLS model.

Viscoelastic descriptors		Lumped parameters
Equilibrium Elastic Modulus	$E_{eq}$	$E_{0}$
Instantaneous Elastic Modulus	$E_{inst}$	$E_{0}+E_{1}$
Characteristic Relaxation Time	$\tau_{rel}$	$\left(\eta_{1}+{\varphi \cdot \eta }_{2}\right)/E_{1}$

The computational pipeline requires a priori knowledge of *c*_*0,*_ $r_{h},\kappa ,\mu ,k_{r}$ and the agarose hydrogel porosity (p) which were either measured or derived (details reported in SI2).

### 2.2 Numerical solving method

The method of lines (MoL) was for the solving the model [60]. Briefly, the 3D spatial domain in Eq. (1) was discretised to replace the partial differential equation (PDE) with a vector system of ordinary differential equations (ODEs) in Cartesian coordinates that can be solved using Matlab ODE45 package (Matlab 2024b, Mathworks.com). The results obtained from the model were used to identify i) the agarose concentrations necessary to obtain hydrogels with an equilibrium elastic modulus matching that of adipose tissue and ii) the liquid phase dextran concentrations required to obtain agarose gels with two Deborah number conditions. The descriptors $E_{eq},E_{inst}$ and $\tau_{rel}$ were derived by solving Eq. (4), performing the non-least square parameter optimisation algorithm.

Further details on the algorithm for solving the model and the values of the extracted parameters are reported in SI1.

### 2.3 Hydrogel preparation for mechanical tests and ADSC culture

To validate the model, gels prepared at different agarose and dextran concentrations were tested using the epsilon dot method (the methods are detailed in SI3). Following the validation, gels matching the equilibrium elastic modulus of adipose tissue (2–6 kPa [45,47,61,62]) were fabricated for cell culture tests using the agarose concentration identified by the computational model (5 mg/mL). The hydrogels were prepared by dissolving agarose powder (A9539, Sigma Aldrich) in sterile deionised water on a magnetic stirrer at 100 °C. Then, dextran powder (D1037, Sigma Aldrich) was dissolved in the agarose solution to obtain the final concentrations of 20, 30, and 40 mg/mL dextran. These concentrations were indicated by the model to yield substrates with De < 1 (40 mg/mL dextran) and De ∼ 1 (20 and 30 mg/mL dextran). The agarose-dextran solutions were cast in 96-multiwells (Ibidi GmbH, Germany) and crosslinked at 4 °C for 30 min to obtain 1 mm-thick gels. Prior to cell seeding, the wells were sterilized under UV for 30 min. To provide adhesive ligands, 60 μL of a 50 mg/mL solution of Gelatin (type A from porcine skin, Sigma Aldrich) in 1X Phosphate Buffer Saline (PBS) was poured on the gels. Gelatin was thus incubated with the gel for 24 h at 4 °C allowing protein absorption on the gel's top surface without affecting bulk mechanical and permeability properties.

Finally, 200 μL of DMEM (Thermo Fisher) supplied with 10 % v/v FBS (fetal bovine serum, Thermo Fisher) and 0.3 mg/mL dextran (added as a buffer to prevent dextran leaching from the samples, see SI2.2) was poured in each well and incubated for an hour to equilibrate the gels.

StemPro™ Human Adipose-Derived Mesenchymal Stem Cells (ADSC, R7788110, Thermo Fisher, passages 4–9) isolated from human adipose tissue were seeded on the hydrogels (50 000 cells/cm^2^) and cultured for 7 days. ADSC seeded on tissue culture plastic (TCP) coated with the same concentration and volume of adhesive ligands (60 μL, 50 mg/mL gelatin) were used as controls.

#### 2.3.1 Cell immunostaining: stem cell surface protein markers and YAP translocation

We investigated YAP localisation in ADSCs to derive the cytoplasmatic and nuclear distribution according to the different viscoelastic substrates [[25], [26], [27]]. The cells were also stained for CD105 (cell surface receptor endoglin) as a positive marker of ADSC stemness and CD45 (a receptor-linked protein tyrosine phosphatase) as a positive marker of ADSC differentiation [[63], [64], [65]]. Immunostaining and imaging were performed 3 and 7 days after seeding. The samples were rinsed twice with 1X PBS with Ca^2+^/Mg^2+^ (DPBS, Thermo Fisher). Then, cells were fixed using 4 % v/v paraformaldehyde/DPBS solution for 20 min at room temperature. 0.1 % v/v Triton® X-100 (Sigma Aldrich) solution in DPBS was added to the samples for 5 min to permeabilise the cell membranes. A solution of 10 % v/v FBS in DPBS was added to block non-specific binding. The samples were incubated at 4 °C for 24h with the primary monoclonal antibodies (Anti-YAP SAB1404823, Anti-CD45 SAB5700873, Anti-CD105 SAB4700258) and then with the secondary antibodies (Goat anti-mouse AlexaFluor® 647, Goat anti-rabbit AlexaFluor® 488, Goat anti-mouse AlexaFluor® 647 respectively) for 2 h at room temperature. All antibodies were from ThermoFisher.

Cytoskeletal staining was performed with Phalloidin solution (Atto-488 and Atto 505, 1:400 in DPBS, Sigma Aldrich), while nuclear staining was with 4′,6-diamidino-2-phenylindole (DAPI D9542, 1 μg/mL in PBS, Sigma Aldrich). The samples were rinsed at least twice with DPBS after every step of the immunostaining procedure.

#### 2.3.2 Evaluation of immunostaining and ADSC morphological parameters

Images were acquired with a confocal microscope (Nikon A1) and analysed using ImageJ (NIH) to evaluate the morphological parameters in the different culture conditions. First, the number of cells per image was estimated from nuclear counting using the Analyse Particle toolbox. The ROI manager toolbox was used to directly evaluate the cell nuclear area (A_N_), the cytosolic area (A) and perimeter (P), to estimate the Cell Shape Index (CSI, Eq. (5)) providing information on cell roundness. Additionally, the nuclear and cytoplasmatic YAP, the CD45 and CD105 mean gray (GI) values were measured from the ROI manager by default as the sum of the gray values of all the pixels in the selection divided by the number of pixels. Finally, the YAP nuclear/cytosolic ratio (YAP_N/C_, Eq. (6)) which represents the distribution of YAP within the cell during the culture was derived.

(5) $$CSI=\frac{4\pi A}{P^{2}}$$

(6) $$Y{AP}_{N/C}=\frac{\frac{Nuclear\;YAP\;GI}{A_{N}}}{\frac{Cytosolic\;YAP\;GI}{A\;}}$$

#### 2.3.3 Histological differentiation assays

Histological assays for ADSC differentiation were performed after 7 days of culture. Adipogenic differentiation was assessed with Oil Red O staining to identify lipid droplets, chondrogenic differentiation was through staining of glycosaminoglycans (GAGs) with Alcian blue, while osteogenic differentiation was assessed through the staining of calcium deposits with Alizarin Red. All the samples were rinsed three times with PBS and brightfield images were acquired. Then, they were dissolved with 100 % v/v isopropanol solution for 30 min, centrifuged and the supernatants were collected and read with the spectrofluorometer to quantify respectively the concentration of lipids (absorbance reading at 500 nm), calcium (absorbance reading at 400 nm) and GAG deposits (absorbance reading at 605 nm), converting the absorbance units in mg/mL through the respective calibration curves.

#### 2.3.4 Data analysis

Statistical analysis, including data processing and comparisons between experimental data and computational simulations, was conducted using GraphPad Prism through t-tests and ANOVA, with a significance threshold set at an alpha level of 0.05 (95 % confidence). The cell culture samples were collected in triplets for each condition and statistical analysis was performed using ANOVA. For the imaging analysis, ten regions of interest per condition were acquired and analysed with 2-way ANOVA.

## 3 Results

### 3.1 In silico model and mechanical testing

**Fig. 4:**
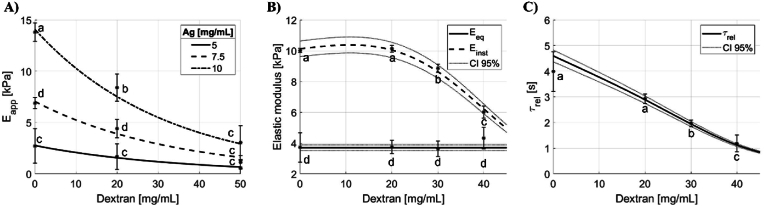
Computed and experimental results as a function of dextran concentration: A) apparent elastic modulus (E_app_) for different agarose concentrations; B) equilibrium (E_eq_) and instantaneous moduli (E_inst_) for 5 mg/mL agarose; C) relaxation time (τ_rel_). 95 % confidence limits are also shown (−). Different letters indicate statistically significant differences in experimental values (p < 0.05).

Fig. 4A reports experimental data and predicted values of the apparent elastic modulus of agarose gels with different concentrations of agarose as a function of dextran concentration. This comparison represents a first validation of the computational framework enabling the selection of agarose concentration (5 mg/mL). This value was used to implement the second computational study to investigate how viscoelastic descriptors vary as a function of liquid phase dextran concentration. The computational model correctly describes the effects of dextran molecules on the viscous components, because only E_inst_ (Fig. 4B) and τ_rel_ (Fig. 4C), are modulated by the dextran-dependent variation of the liquid phase viscosity, while E_eq_ does not change significantly. In fact, E_inst_ and τ_rel_ decrease with increasing dextran concentration as the gel becomes more liquid-like, in coherence with the dextran-dependent dashpot arm of the aSLS model (Fig. 4B). Further computational investigations, reported in the SI4, show that the apparent diffusion coefficient (D_app_) and mesh size (ξ_avg_) increase as the dextran concentrations increase (Fig. S6A and S6B). On the other hand, the liquid phase diffusion time (τ_diff_) decreases with increasing concentrations of dextran molecules in the system (Fig. S6C). Further details, including the extracted lumped parameters and viscoelastic descriptors and the comparison between the stress-time curves predicted and experimentally recorded (Fig. S5), are reported in the SI1 and SI3 respectively.

### 3.2 Confirmation of the design approach

Considering that τ_b_∼1s for ADSC and that τ_L_ varies between a minimum and maximum value of 10 and 100 s [3,11] as reported in the literature, we were able to match the conditions of De < 1 and De∼1 by varying the dextran concentration in the gel liquid phase. In particular, the gels with a dextran concentration of 40 mg/mL give a De < 1 (0.01) with τ_rel_ ≤ τ_b,_ thus the material relaxes before the cell binding processes are complete. On the other hand, dextran concentrations of 30 or 20 mg/mL result in De values near 1 (0.2 and 0.3, respectively), representing the case in which material viscoelastic dynamics occur at around the same timescales as cell binding processes. To decouple the effects of varying dextran concentration from changes in De condition, we selected both 30 and 20 mg/mL of dextran to represent the De ∼1 condition. Considering that the TCP controls have a theoretically infinite relaxation time (since τ_rel_ → ∞, De = τ_rel_/τ_L_ → ∞), they could represent the condition De > 1, which promotes stable FAs. However, cell responses on TCP controls cannot be compared with the agarose gels, because - albeit the adhesive ligands are the same (gelatin coating)- the substrate chemistry is quite different.

**Fig. 5:**
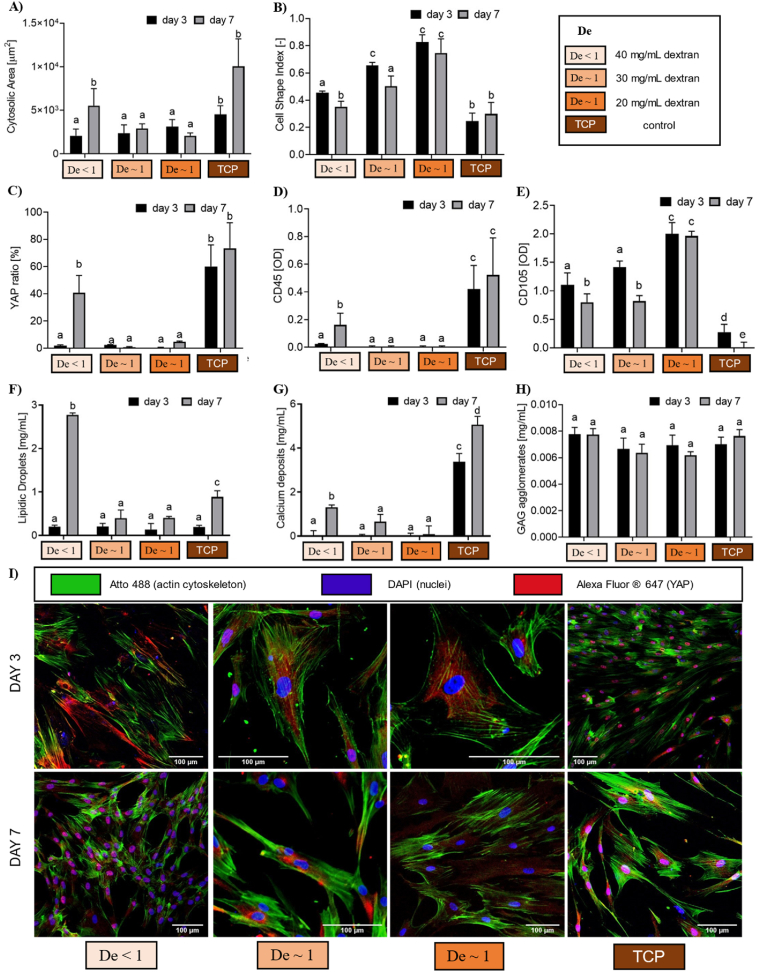
Morphological, immunostaining and histological analysis as a function of the substrate Deborah number condition: A) ADSC cytosolic area, B) cell shape index (CSI), C) nuclear vs cytosol YAP ratio, D) CD45^−^and E) CD105-associated protein quantifications (unit: optical density, OD), Quantification of F) lipidic droplets (Oil Red O), G) calcium deposits (Alizarin Red) and H) GAG agglomerates (Alcian Blue). Different letters indicate statistical significance (p < 0.05). Controls are TCP. I) YAP immunostaining on ADSC after 3 and 7 days of culture on the agarose substrates and on the TCP controls. Cell nuclei are stained in blue (DAPI), actin cytoskeleton in green (Atto-488) and YAP molecules in red (Alexa Fluor® 647). (For interpretation of the references to colour in this figure legend, the reader is referred to the Web version of this article.)

Fig. 5 reports the immunostaining analyses and histological results for ADSCs cultured on the selected substrates, highlighting how the combination of substrate $\tau_{rel}$ with the ADSC sensing timescales enables either differentiation or the maintenance of stemness. At day 7, ADSCs cultured on De ∼1 hydrogels exhibit a significantly smaller cytosolic area (Fig. 5A) and a higher CSI (Fig. 5B), retaining a rounded morphology typical of stem cells with respect to the De < 1 gel and TCP control. Notably, Fig. 5C and I reveals that the nuclear-to-cytoplasmic YAP_N/C_ ratio is nearly zero on the De ∼1 substrates, with YAP entirely localized in the cytoplasm maintaining ADSCs in an undifferentiated, stem-like state. In contrast, ADSCs on the De < 1 hydrogel and TCP control show an increased cytosolic area and lower CSI. After seven days of culture, YAP translocates into the nucleus on the De < 1 gel, with YAP_N/C_ ratio values statistically comparable to TCP controls where the translocation occurs from day 3. This response is further corroborated by membrane protein expression profiles associated with CD45 and CD105. Fig. 5D shows a high expression of CD45 markers in ADSCs on TCP controls, indicating a loss of stemness at both day 3 and 7. By day 7, CD45 markers are also observed in ADSCs cultured on De < 1 gels but are absent on De ∼1 gels. Conversely, Fig. 5E shows that CD105 markers, absent on TCP controls, are highly expressed in ADSCs on De ∼1 substrates.

Histological assays support these findings, showcasing variations in differentiation across substrates. ADSCs on TCP controls undergo osteogenic differentiation by day seven, evidenced by calcium deposits in the Alizarin Red assay (Fig. 5G). Differently, Oil Red O staining (Fig. 5F) reveals lipid droplets in ADSCs on De < 1 gels. The Alcian Blue assay, shown in Fig. 5H, did not identify any differences in GAG production in the investigated dataset. Additional information, including confocal scans of CD45 (Fig. S7) and CD105 (Fig. S8) and brightfield images from histological staining (Fig. S9), are reported in SI5.

## 4 Discussion

In the dynamic landscape of mechanotransduction studies, a strategy for the precision design of substrates aimed at aligning hydrogel viscoelasticity with cellular timescales is lacking. Establishing how cells interpret a substrate is crucial to control their stemness or direct their differentiation. However, most studies in the literature limit their investigations to observations of cell behaviour in response to substrate viscoelastic properties, without considering the disparity between cellular timescales and material relaxation times [[2], [3], [4], [5], [6], [7], [8], [9]]. Here we describe a novel design approach, which allows prediction of gel compositions with precise viscoelastic features able to elicit specific cell responses on the basis of their sensing timescale. By introducing the cell-perceived Deborah number (De), which explicitly links substrate relaxation time to cell adhesion timescales, we defined a metric that enables the rational design of biomaterials that match cellular mechanotransduction specifications.

We then developed a computational workflow to predict the viscoelastic behaviour of dextran-permeated agarose hydrogels and allow the selection of substrates with relaxation times matching the timescales typical of cellular adhesion processes. Our model shows that the agarose mesh size and dextran diffusion coefficient increase with increasing concentrations of dextran, and therefore that mass transport of dextran through the polymer network is facilitated. In fact, although dextran increases the viscosity of the liquid phase (Fig. S4A, SI2), its presence also increases liquid phase mobility and reduces resistance to flow. This is also reflected in the viscoelastic properties of the system, which becomes more liquid-like (decreasing τ_rel_) with increasing dextran concentrations. These results support our initial hypothesis that dextran hinders water-agarose interactions [10,31]. Moreover, the addition of η_2_ in the aSLS model was effective for modelling the contribution of dextran to the viscous behaviour of the dextran-penetrated gels. In fact, the dextran independent viscous element, η_1_ can be considered to represent the combined effect of both water viscosity and the dissipative behaviour of agarose (chain entanglements and molecular friction), while E_1_ represents the system's instantaneous response, before internal friction effects occur. Finally, E_0_ (i.e., E_eq_), which is related to the equilibrium elastic behaviour of the system, after viscous phenomena are completely dissipated, is not affected by the presence of dextran. In summary, the proposed modelling framework provides a simple method to predict hydrogel viscoelastic properties with input parameters that can be easily derived from literature or extrapolated with standard laboratory facilities, namely the liquid phase viscosity, the hydrodynamic radius and the hydrogel porosity. This enables formulating a target hydrogel composition as a function of the addressed cell timing with minimal experimental costs and time.

Many existing models for describing hydrogel viscoelasticity rely on complex finite element methods or energy balance approaches, which can be computationally expensive [5,8,[32], [33], [34],^66^]. In contrast, our model is based on experimentally derived parameters (Figs. S2 and S4), providing a rapid and easily implementable tool to predict gel viscoelastic features. A key distinction of our model compared to existing approaches lies in its computational efficiency and the integration of structural and transport properties. Integrating diffusive and porosity-related parameters with mechanical descriptors thus allows the prediction of hydrogel behaviour in two physics regimes.

Although the model was specifically developed to describe the interaction between dextran molecules and agarose network, the framework can be extended to other polymers with the same advantages. Future refinements – particularly for materials which unlike agarose, tend to manifest significant spatial gradients in porosity and viscosity – may also include the potential role of anisotropy and inhomogeneities in the polymer network. This would require a detailed characterization of the gel microstructure through imaging techniques or microrheological experiments. Refinements of this nature would also be relevant in studies on graded structures for mimicking tissue interfaces. Furthermore, the model may overlook complex molecular interactions or structural adaptation phenomena over time. This could be critical for some material combinations, where dynamic interactions or non-linear effects might occur. In such cases, improvements in model's accuracy, such as the integration of more complex mechanical models based on higher lumped parameter representations, poroelastic models or Prony series [10] or incorporating multi-scale simulations and machine learning for adaptive parameter estimation [24,32,34,66], along with broader experimental validation, could be implemented.

After confirming the predictivity of the in silico model with experimental data from mechanical tests, the efficacy of the design approach for dextran-permeated agarose gels was demonstrated through the study of mechanotransduction in ADSC. The model was used to select substrate compositions capable of directing ADSC behaviour, investigating morphological, immunological and histological outcomes to probe how substrate viscoelasticity affects cell response as a function of the relationship between substrate relaxation time (τ_rel_) and the cellular sensing time window, using the cell-perceived Deborah number, De. Since ADSCs have been widely characterised, in this work we used literature values for FA binding- and lifetime. The De-based design strategy proved to be effective, nonetheless, a direct measurement of FA dynamics using traction force microscopy may improve the precision of the design process.

The De was crucial in the maintenance of cell stemness or differentiation. Substrates with De∼1 maintain ADSC stemness as shown by the expression of stemness-related CD105 markers (Fig. S7), and the fact that YAP proteins were fully localised in the cytoplasm [45,65]. In these conditions, the cells perceive a mechanically time-varying environment which prevents the formation of stable FAs that hinder differentiation, resulting in mechanical conditions similar to those of the adipose niche. Instead, both the De < 1 gel and the TCP control substrates elicit differentiation in cells. In these conditions, mechanical stimuli may in fact produce the tension required for YAP transmigration into the nucleus along the actin fibres, kicking off the pathways for differentiation. Additionally, the expression of CD45 markers (Fig. S8), which are strongly expressed in differentiated cells, supports the differentiation process hypotheses [25,27,45]. TCP can be modelled as a purely elastic material with τ_rel_→∞ and an elastic modulus up to 10 GPa. Since the average focal adhesion lifetime is between 10s and 100s [11,21], culturing ADSCs on TCP corresponds to a case in which De > 1, which means that, from a cell perception point of view, the substrate is mechanically stable as its stiffness is practically time-invariant. As a result, cells seeded on TCP encounter an almost static mechanical environment with a high stiffness that results in constant high stress on the cytoskeleton. This effect promotes cell spreading and YAP transmigration, leading to differentiation towards the osteogenic phenotype, as shown by the Alizarin Red assay (Fig. S9). In the case of 5 mg/mL agarose substrates with 40 mg/mL dextran, τ_rel_ is close to 1 s and De < 1. Since τ_b_ ≈ 1s, the viscoelastic dynamics evolve rapidly and are over before FA are formed. In this case, cells sense a constant (but low) equilibrium stress promoting FA stabilisation. Since the final sensed stiffness (E_eq_) is equal to the elastic modulus of adipose tissue, the combination of the elastic and viscous components determines the mechanical stimulation required to induce ADSC adipogenic differentiation (as indicated by the Oil Red O assay in Fig. 5F and Fig. S9) [27,46,67,68].

**Fig. 6:**
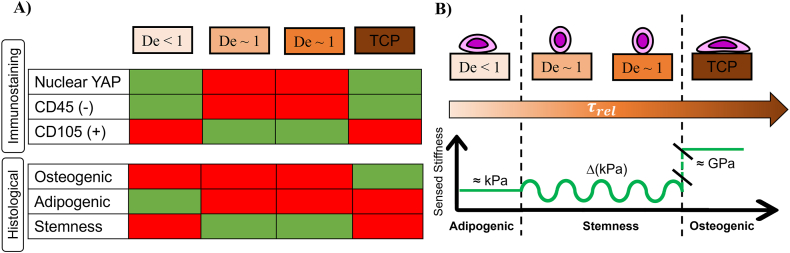
A) Summary of histological and immunostaining profiles of ADSC in the mechanotransduction study. Colour legend: Red/Green = absence/expression of the specific markers and/or respective deposits at the end of the cell culture. B) Schematic drawing representing the influence of the combining substrate stiffness and time-dependent properties in guiding ADSC stemness or differentiation. (For interpretation of the references to colour in this figure legend, the reader is referred to the Web version of this article.)

In summary, the perception of a mechanical stimuli changing over time prevents FA stabilisation, promoting ADSC stemness (De∼1). Instead, when the dynamics of the stimuli are outside of the sensing window, the perceived mechanical signals are stable and promote cell differentiation according to intensity of the mechanical cue - here, osteogenic and adipogenic differentiation respectively for stiffer and softer substrates. Fig. 6 illustrates how the relationships between substrate stiffness and time-dependent properties guide ADSC stemness or differentiation.

## 5 Conclusions

We propose a novel computational approach for the accurate design of hydrogels with tunable viscoelastic properties for matching cellular timescales. Through the definition of a cell-perceived Deborah number, our approach allows tailoring substrates to match cell sensing times, thus inducing cell differentiation or preserving their stemness. This result is of considerable impact in the field of substrate manufacturing aimed at preserving cell stemness in a niche-like environments, without resorting to complex biochemically-engineered artificial niches [[42], [43], [44],^69^].

Besides including different material combinations as well as different cell types, in the future, the approach's versatility might be increased by integrating it with a computational representation of cell-material interaction (e.g., the well-established molecular clutch model) to provide an all-in-one tool for the selection of the appropriate substrates in terms of viscoelastic features able to maintain cell stemness or promote their differentiation toward a specific lineage. Advancements in this research direction will be critical for the development of advanced physiological and pathological in vitro models with more human-relevant outcomes in *in vitro* studies, regenerative medicine applications, or for more in depth mechanobiology investigations.

## CRediT authorship contribution statement

**Nicole Guazzelli:** Writing – original draft, Investigation, Data curation. **Ludovica Cacopardo:** Writing – original draft, Methodology, Investigation, Conceptualization. **Arti Ahluwalia:** Writing – original draft, Methodology, Conceptualization.

## Funding

This research received funding from the 10.13039/501100003196Italian Ministry of Health for the development of alternatives to animal-based research.

## Declaration of competing interest

The authors declare that they have no known competing financial interests or personal relationships that could have appeared to influence the work reported in this paper.

## Data Availability

Data will be made available on request.
