# Supplementary material for: Precision design of dextran-permeated agarose hydrogels matching adipose stem cell adhesion timescales
Source: Mater Today Bio. 2025 May 6;32:101832. doi: 10.1016/j.mtbio.2025.101832 (PMC12145710; doi:10.1016/j.mtbio.2025.101832)
Supplement: Multimedia component 1 [file mmc1.docx]

Supplementary Information

Precision design of dextran-permeated agarose hydrogels matching adipose stem cell adhesion timescales

Nicole Guazzelli* ^1,3^, Ludovica Cacopardo* ^1, 2, 3^, Arti Ahluwalia ^1,2, 3^

**S1. Parameter identification through Matlab - Simulink comparison**

The system function of the aSLS model (Eq. 1) was implemented on Simulink 10.5 (Figure S1A) to simulate the stress – time response as a function of a constant input strain rate ($\dot{\varepsilon}$). The presence of dextran was modelled with the bias block: if the bias is equal to zero, the model solves the lumped parameter schematization without the second dashpot, otherwise, if the bias is equal to 1, the model also considers the effect of dextran through the second dashpot weighted according to the volumetric fraction of dextran.

| $\dot{\sigma}+ \frac{E_{1}}{\eta_{1}} \sigma=\left( E_{0}+ E_{1} \right)\dot{\varepsilon}+ \frac{E_{0}\cdot E_{1}}{\eta_{1}+{\varphi_{dex}\eta}_{2}} \varepsilon$ | (Eq.1) |
| --- | --- |

The lumped parameters were identified by performing an iterative parameter identification algorithm (Figure S1B). Simulink was used to generate the stress – time curve ($\sigma_{Sim}(t)$) by setting all lumped parameters to a value of 1. In the next step, the $\sigma_{Sim}(t)$ curve was optimised to fit the stress – time curve ($\sigma_{Mat}(t)$) obtained from Matlab by adjusting the lumped parameters using a least square minimisation algorithm. The parameter adjustments were made while adhering to the constraints on their values, as detailed in in Table S1.

Table S1. Constraints and intervals used in the numerical solving approach for lumped parameter identification.

| **Lumped parameter** | **Constraints** | **Unit** |
| --- | --- | --- |
| E_0_ | [1,10^5^] | Pa |
| E_1_ | [1,10^6^] | Pa |
| η_1_ | [1,10^10^] | Pa·s |
| η_2_ | [1,10^10^] | Pa·s |
| **Interval** | **Value** |  |
| dη | 1 | Pa·s |
| dE | 1 | Pa |

To reduce the computational time and avoid over-parametrisation, the first optimisation was performed on a simple SLS lumped parameter model representing only agarose, without considering the additional piston η_2_ to identify E_0_ and η_1_. Then dextran was introduced keeping E_0_ and η_1_ fixed so as to reduce the number of parameters to be identified.


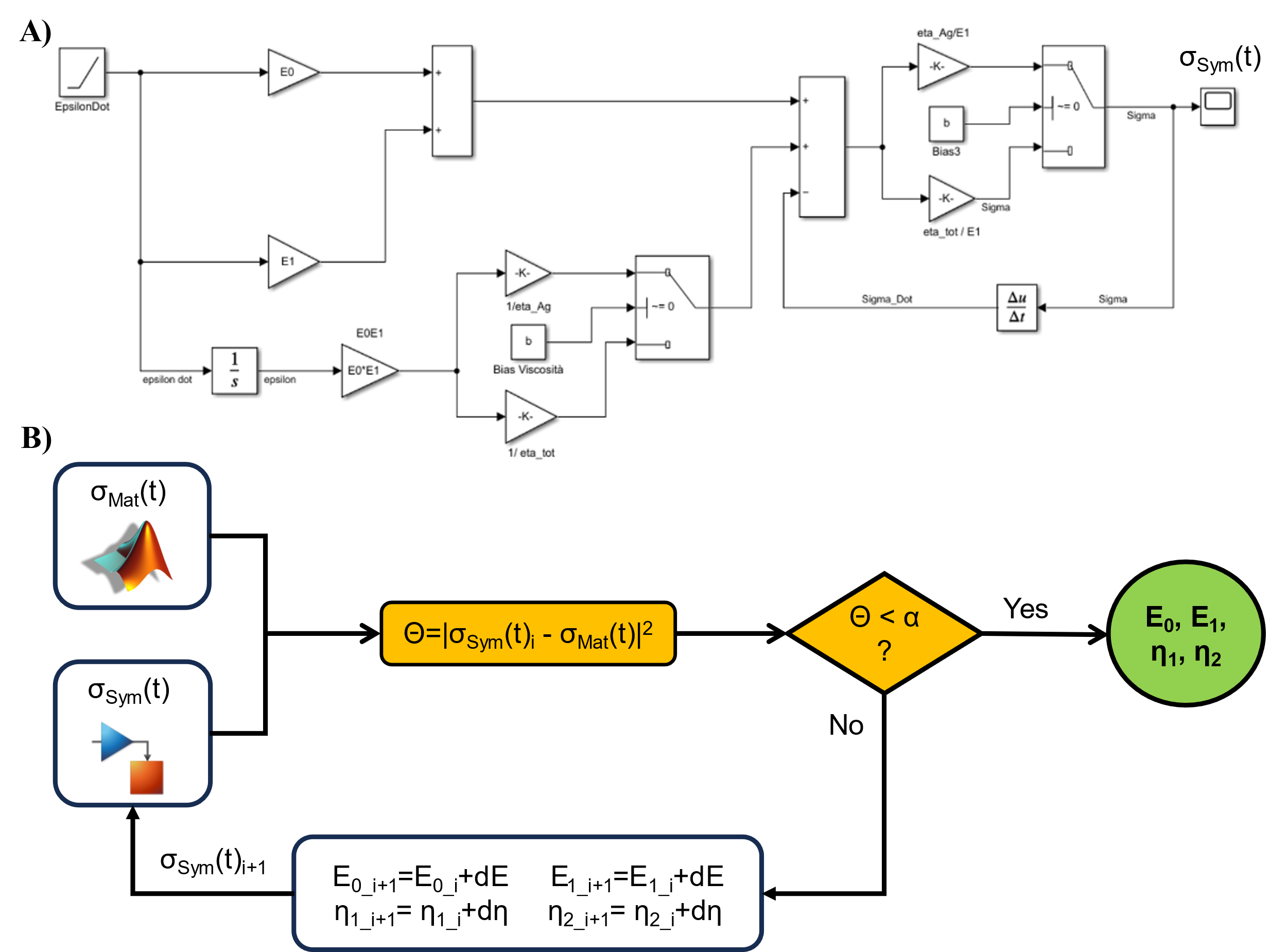


Figure S1. A) aSLS block diagram architecture for simulating the mechanical behaviour of dextran-agarose hydrogels during a compression test according to the epsilon dot method^1–3^; B) Diagram of the iterative optimisation algorithm for the identification of lumped parameters.

Table S2 reports the values of the lumped parameters, and the related viscoelastic descriptor derived from the in silico model in the case of 0. 20, 30 mg/mL dextran corresponding to values of De $\sim$ 1, and 40 mg/mL dextran corresponding to a value of De < 1.

Table S2. Values of the lumped parameters and viscoelastic descriptors estimated from the in silico framework for the dextran concentrations used in the experimental validation.

|  | **Dextran [mg/mL]** | | | |
| --- | --- | --- | --- | --- |
|  | **0** | **20** | **30** | **40** |
| **E_0_ [Pa]** | 3.74E+03 | 3.74E+03 | 3.74E+03 | 3.74E+03 |
| **E_1_ [Pa]** | 6.28E+03 | 6.19E+03 | 4.52E+03 | 2.27E+03 |
| **η_1_ [Pa·s]** | 2.76E+04 | 2.76E+04 | 2.76E+04 | 2.76E+04 |
| **η_2_ [Pa·s]** | 0.00E+00 | 4.44E+05 | 6.02E+05 | 6.13E+05 |
| **E_eq_ [Pa]** | 3.74E+03 | 3.74E+03 | 3.74E+03 | 3.74E+03 |
| **E_inst_ [Pa]** | 1.01E+04 | 1.00E+04 | 8.56E+03 | 6.31E+03 |
| **τ_rel_[s]** | 4.39E+00 | 3.02E+00 | 2.10E+00 | 1.34E+00 |
| **De** | 0.44($\sim$1) | 0.30($\sim$ 1) | 0.21($\sim$ 1) | 0.01(< 1) |

**SI2. Evaluation of the in silico model input parameters**

*S2.1. Evaluation of water-dextran binding rate*

The water-dextran binding rate $k_{r}$ was determined by analysing the viscosity measurements of dextran aqueous solutions at a concentration of 20 and 40 mg/mL, across a range of temperature (from 60°C to 25°C). The data were fitted using a least-squares minimization algorithm applied to the generalised Arrhenius equation^4^ (Eq. 2). The calculation of $k_{r}$ followed the formulation outlined in Eq.3, where $A$ represents the pre-exponential factor ([Pa·s])^4^, $t$ represents the duration of the viscosity acquisition (180 s), $\varphi_{dex}$ the dextran volumetric fraction, and $\mu_{ref}$ denotes the reference viscosity of water, set at 1.06·10^-2^ Pa·s ^5^. The optimisation yielded a $k_{r}$ value of (2.39 ± 0.186)·10^-4^ s^-1^ (Figure S2).

| $\mu\left( T \right)=A\cdot{exp}^{\left( -\frac{b}{T} \right)}$ | (Eq.2) |  |  | $k_{r}=\frac{A}{\varphi_{dex}\cdot t\cdot\mu_{ref}}$ | (Eq.3) |
| --- | --- | --- | --- | --- | --- |


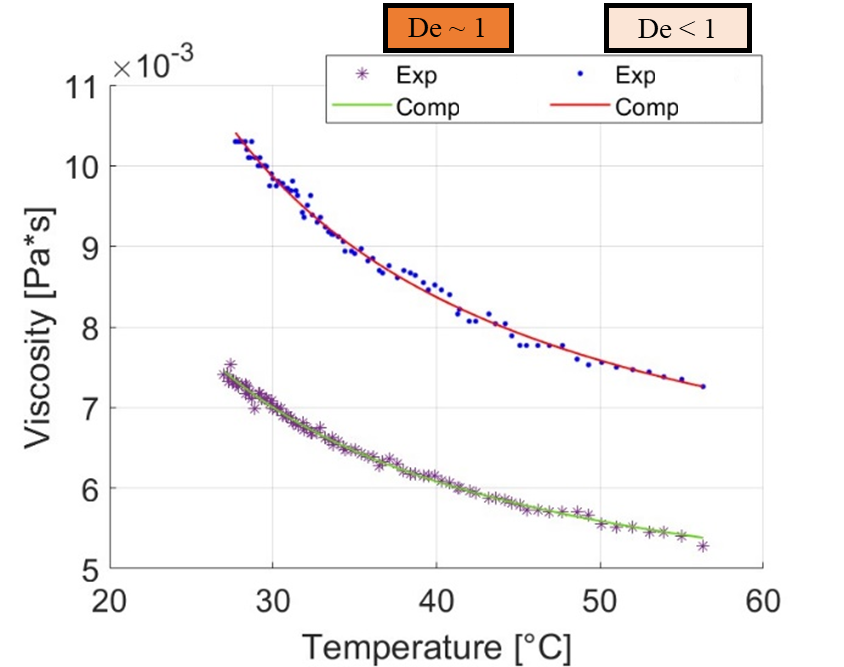


Figure S2. Experimental and computational trend of the liquid phase viscosity in case of De ~ 1 and De <1 (respectively, 20 and 40 mg/mL dextran) as a function of the temperature (A = 10^5^).

*S2.2. Estimation of dextran hydrodynamic radius*

The hydrodynamic radius $r_{h}$ of dextran molecules was estimated using an integrated experimental and in-silico approach. First, 13x13x5 mm slabs of 5 mg/mL agarose hydrogels prepared with 50 mg/mL fluorescein isothiocyanate conjugate-dextran (FITC-dextran, 500 kDa, 46947 Sigma-Aldrich) were lodged in a purposely machined rectangular chamber, twice the volume of the gel (Figure S3A). After adding deionised water to the empty region of the chamber, the diffusion of FITC-dextran from the gel to the liquid region was measured by fluorimetry 1, 4 and 24 hours, keeping the system at 37^◦^C. Then a 2D-axial symmetric time-dependent FEM simulation of dextran diffusion from the agarose hydrogel was run on Comsol Multiphysics 6.2 (Figure S3A), solving the transport of diluted species in porous media with the reaction between dextran and water. Based on the data from Hagman et al.^6^, $r_{h}$was considered to lie in the range of 12.05 and 25.92 nm. The computational results were optimised within this range to fit the measured concentration- time data using a least square minimisation algorithm considering 95% confidence limits. The best prediction of the diffused dextran concentration outside the gel was for $r_{h}$= (22 ± 4.67) nm as reported in Figure S3B.


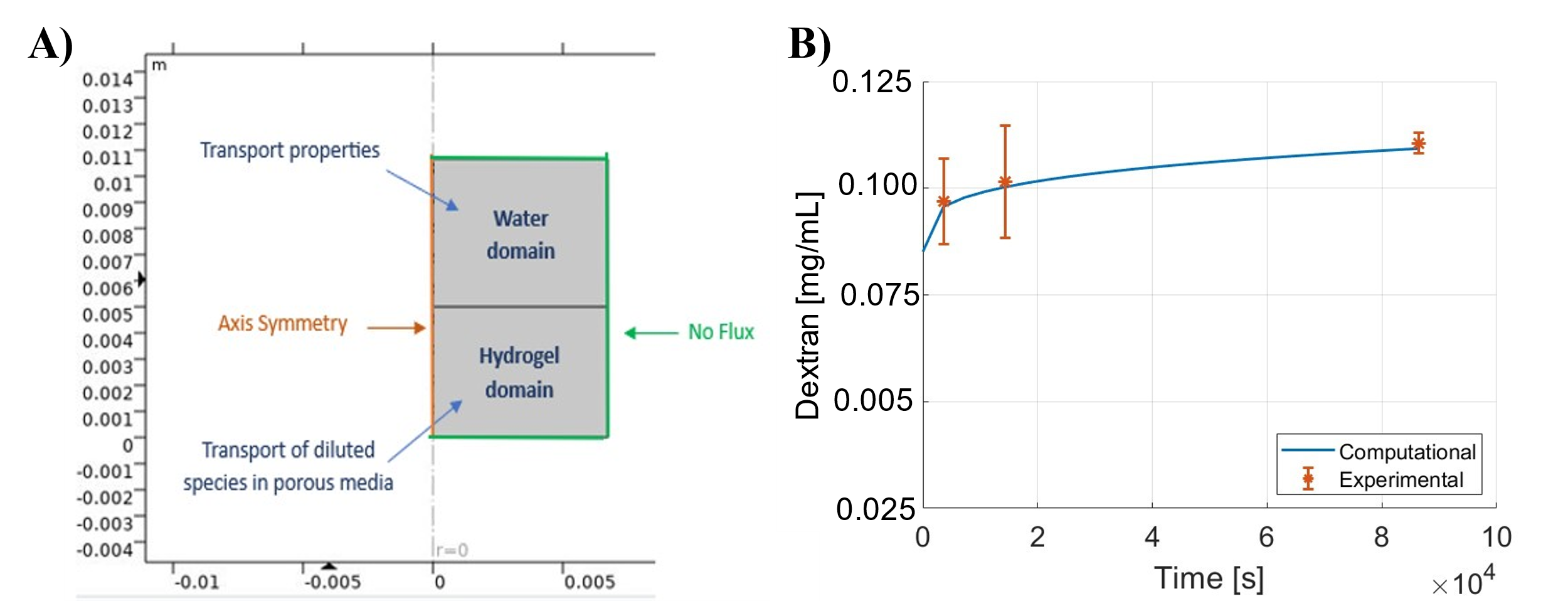


Figure S3. A) FEM 2D-axial symmetric domains geometry and B) comparison of the experimental and computational FITC-dextran concentration trend in time for 5 mg/mL agarose hydrogels.

*S2.3. Measurement of liquid phase viscosity and derivation of hydrogel porosity*

The dynamic viscosity µ of the dextran solutions (20, 30 and 40 mg/mL dextran in deionised water, D1037, Sigma Aldrich) was measured using a dynamic viscometer (DV-II+, Brookfield, Germany) as a function of the temperature; the LV1 spindle was chosen considering the range of viscosity values (3·10^0^ - 6·10^5^ cP).

Swelling tests were performed to derive the agarose hydrogel porosity according to the Archimedes fluid motion principle^7^. 5 mg/mL agarose powder (A9539, Sigma Aldrich) was dissolved in deionized water at 100°C. Then, dextran powder in different concentrations (0, 20, 40 mg/mL, D1037, Sigma Aldrich) was dissolved in the agarose solution. The solutions were cast into custom cylindrical moulds (diameter 13 mm, height 8 mm). After thermal crosslinking at 4°C for 30 minutes, the samples were dried at 50 °C for 24h. Each dried sample was submerged in 3 mL of deionised water and the changes in gel weight, diameter and height were collected after different time points (30 minutes, 1, 1.5, 2, 2.5, 3 and 24 hours). The hydrogel porosity was derived from the dried ($V_{d}$) and the swollen ($V_{s}$) volumes (Eq.8).

| $p= \frac{V_{pores}}{V_{tot}}*100= \frac{V_{s}-V_{d}}{V_{s}}*100$ | (Eq.8) |
| --- | --- |

The experimental values of viscosity (Figure S4A) and porosity (Figure S4B) were fitted with custom fitting curves (respectively $\mu=ax^{b}+c$ and $p=\frac{ax}{b+x}+c$) and the nonlinear least square Levenberg-Marquardt algorithm (Matlab 2023a) to define the auxiliary functions recalled in the Simulink model workspace. The specific fitting laws were used to consider other material trends, widening the applicability of the model.


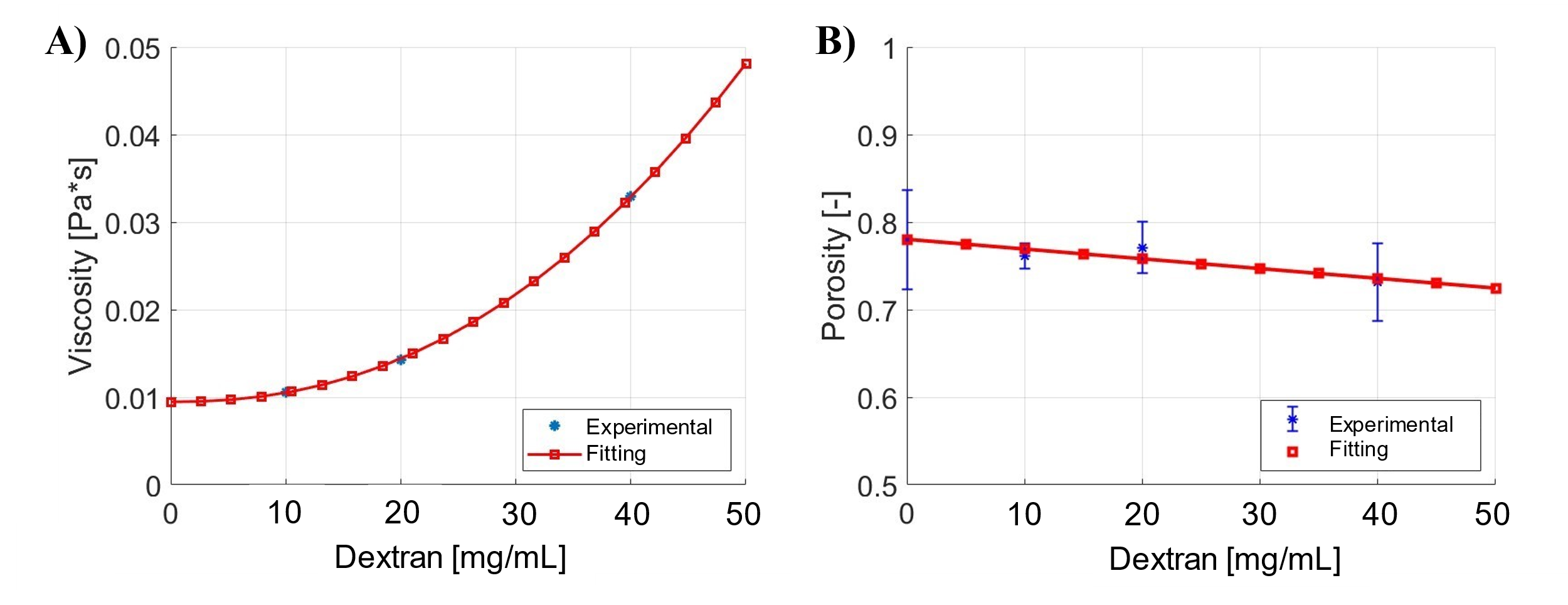


Figure S4. Experimental data and corresponding fits for A) Liquid phase viscosity and B) hydrogel porosity for different dextran concentrations permeating 5 mg/mL agarose hydrogels. The fitted equations were recalled as auxiliary functions in the algorithm shown in Figure S1 (fitting coefficients: Viscosity: a = 52.885, b = 2.412, c = 0.010, R^2^ = 0.991 – Porosity: a = -1.392e+05, b = 1.141e+05, c = 0.785, R^2^ = 0.998).

Table S3 reports the boundary conditions, and the discretised time and spatial steps implemented in the computational model.

Table S3. Boundary conditions for solving the reaction-diffusion equations on Matlab with MoL

| **Boundary and step conditions** | **Symbol** | **Values** | **Unit** |
| --- | --- | --- | --- |
| Initial position | $\bar{s}_{0}$ | 0 | m |
| Final position | $\bar{s}_{f}$ | 1 ·10^-3^ | m |
| Geometric step | ds_i_ | 1·10^-5^ | m |
| Time step | dt | 1·10^-2^ | s |
| Upper concentration boundary | c_U_ | 0 | mol/m^3^ |
| Lower concentration boundary | c_L_ | c_0_ | mol/m^3^ |

*S2.4. Liquid phase permeability estimation*

The liquid phase permeability κ was estimated through the Carman Kozeny equation (Eq.4) as a function of the gel porosity *p*, the dextran hydrodynamic radius r_h_ and the Kozeny factor *k* (Eq.5). The latter is related to the channel shape and tortuosity; it is derived as a combination of a parallel ${(k}_{\parallel} )$ (Eq.6) and perpendicular ${(k}_{\perp})$ (Eq.7) components with respect to the flow coordinates^8^.

| $\kappa=\frac{pr_{h}^{2}}{k}$ | (Eq.4) |
| --- | --- |
| $k=\frac{(2k_{\perp}+k_{\parallel})}{3}$ | (Eq.5) |
| $k_{\parallel}=\frac{2p^{3}}{\left\{ \left( 1-p \right)\left[ ln \left( \frac{1}{1-p} \right) -3+4\left( 1-p \right)-\left( 1-p \right)^{2} \right] \right\}}$ | (Eq.6) |
| $k_{\perp}=\frac{2p^{3}}{\left\{ \left( 1-p \right)\left[ ln \left( \frac{1}{1-p} \right) -\frac{1-\left( 1-p \right)^{2}}{1+\left( 1-p \right)^{2}} \right] \right\}}$ | (Eq.7) |

**SI3. Hydrogel fabrication and characterisation for model validation**

To validate the predictive capability of the model, we generated an experimental dataset by deriving the viscoelastic properties of agarose-dextran hydrogels under controlled mechanical testing conditions. Dextran solutions were prepared dissolving the dextran powder (D1037, Sigma Aldrich) at concentrations of 0, 20, 30 and 40 mg/mL in deionised water on a magnetic stirrer. Agarose-dextran hydrogels were prepared by dissolving different concentrations (5, 7.5, 10 mg/mL) of agarose powder (A9539, Sigma Aldrich) in the dextran solutions on a magnetic stirrer at 100°C. The agarose-dextran solutions were cast in custom-made PDMS moulds (cylindrical wells: 13 mm diameter, 8 mm height) and crosslinked at 4°C for 30 minutes to obtain the agarose-dextran hydrogels. The gel viscoelastic parameters were estimated using the Epsilon-dot method^9^. Briefly, unconfined bulk compression tests at different constant strain rates (=0.0005, 0.001, 0.005, 0.01 s^-1^) were performed using the Zwick/Roell ProLine Z005 uniaxial testing device (Germany). Each sample was tested under hydrated conditions. The time-stress curves were collected and globally fitted to the aSLS equation (Eq.3) to obtain the values of the lumped parameters $E_{0},E_{1},\eta_{1}, \eta_{2}$, and thus the descriptors E_inst_, E_eq_ and τ_rel_. More specifically, the global fitting procedure was applied to the experimental time-stress curves from gels of the same composition measured at different strain rates, in accordance with the Epsilon-dot method ^9^. This allowed robust estimation of the viscoelastic parameters across varying loading conditions. Moreover, the apparent elastic modulus ($E_{app}$) was estimated from the slope of the stress-strain curves in the linear viscoelastic region (LVR, 5%). The curve fitting process, the comparison between the experimental results and the computational predictions, as well as the statistical 1-way ANOVA and t-tests analyses were performed on OriginPro 2015 and GraphPad Prism to determine the significance of the results and assess the predictive power of the in silico framework. The validation dataset and model outputs are in the main text (Figure 4) while Figure S5 provide a comparison between experimental stress-time curves (red lines) and computational model predictions (blue lines) for agarose hydrogels containing 0, 20, 30, and 40 mg/mL dextran. The curves refer to a single strain rate of 0.01 s⁻¹ and represent the Deborah number conditions used in the cell culture experiments. This figure is not related to the global fitting procedure, which was instead performed across multiple strain rates for each composition to extract the viscoelastic parameters used to validate the model.


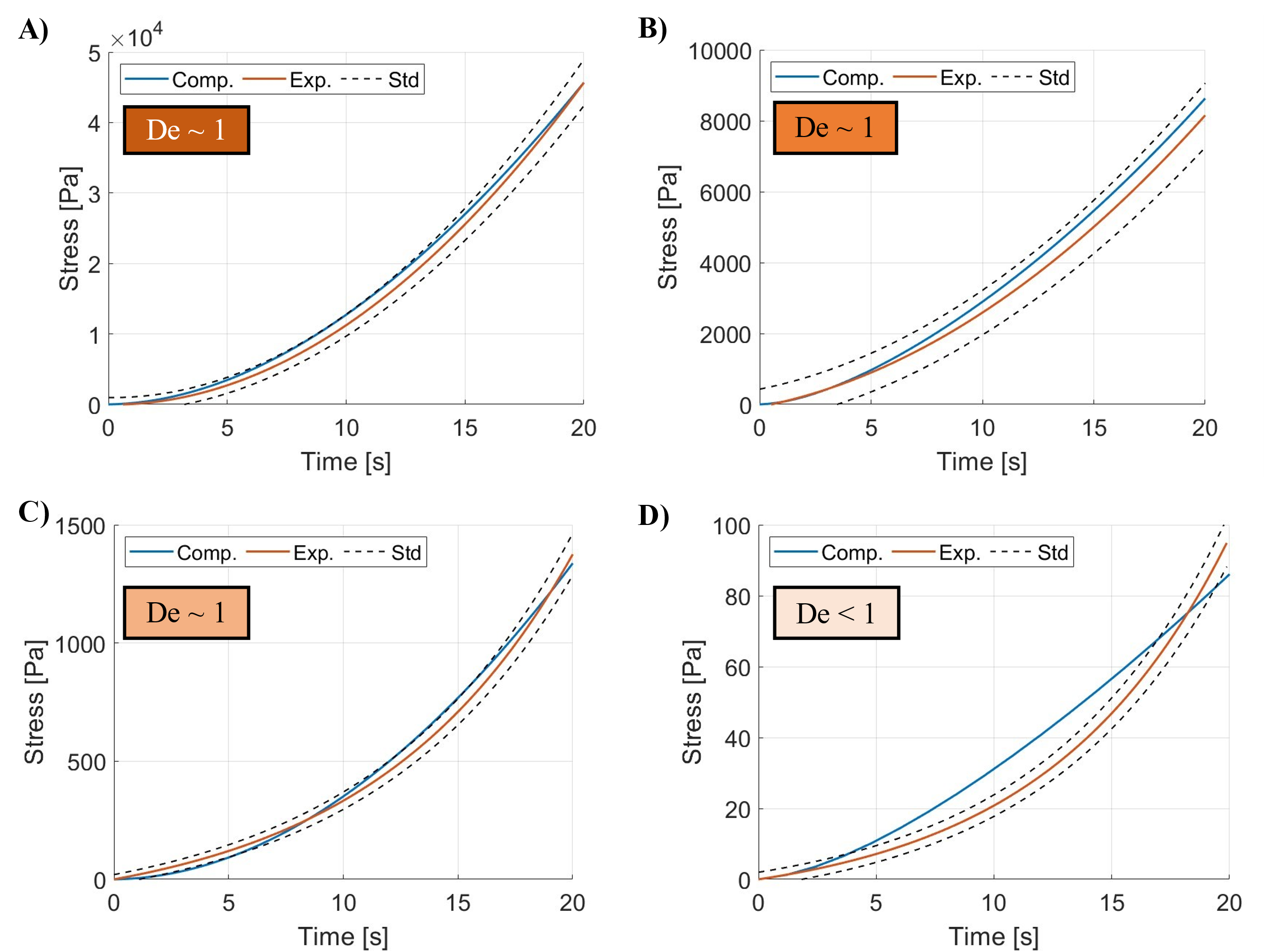


Figure S5. Comparison between the predicted (blue lines) and measured (red lines) stress-time curves in the case of agarose gel with 0 mg/mL (A), 20 mg/mL (B), 30 mg/mL (C) and 40 mg/mL (D) dextran considering a strain rate of 0.01 s^-1^.

**SI4. Prediction of diffusive properties**

Figure S6 reports the computational prediction of the diffusive properties, i.e., the diffusion coefficient, the average mesh size and the characteristic diffusion time as a function of different agarose and dextran concentrations.


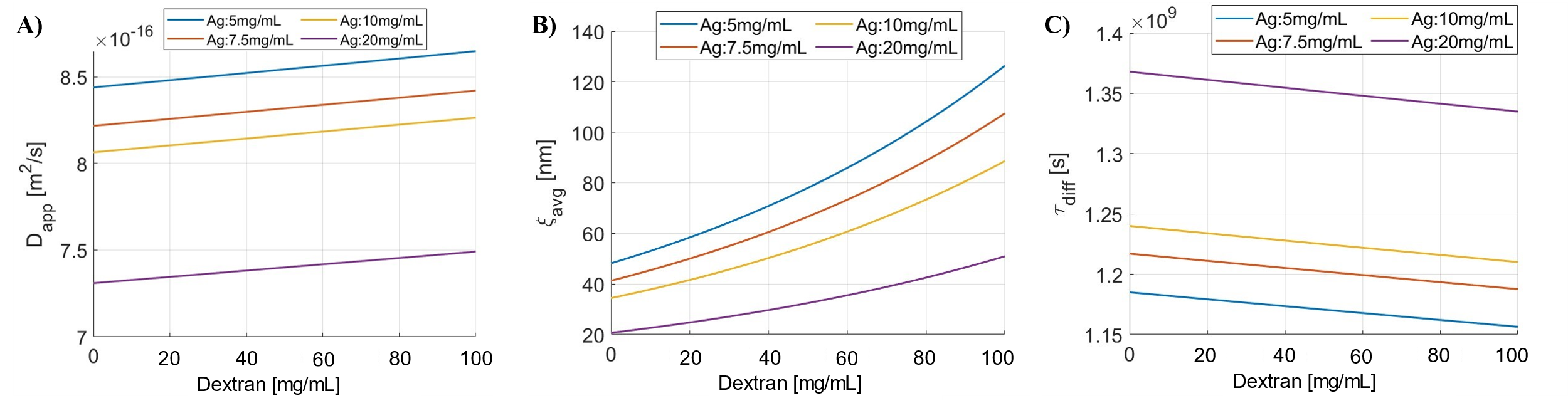


Figure S6. Computational prediction of the: A) apparent diffusion coefficient; B) mesh size; C) diffusion time as a function of dextran concentration for different agarose hydrogels.

**SI5. ADSC viscotransduction study: immunostaining and histological staining**

Figures S7 and S8 report respectively the confocal results of the immunostaining for CD105 and CD45 associated protein markers after 3 and 7 days of ADSC culture for different substrates, referred to on the basis of the cell-perceived Deborah number De. Figure S9 reports the brightfield images following histological staining after 7 days of ADSC culture for the identification of the differentiation lineages for different De.

*
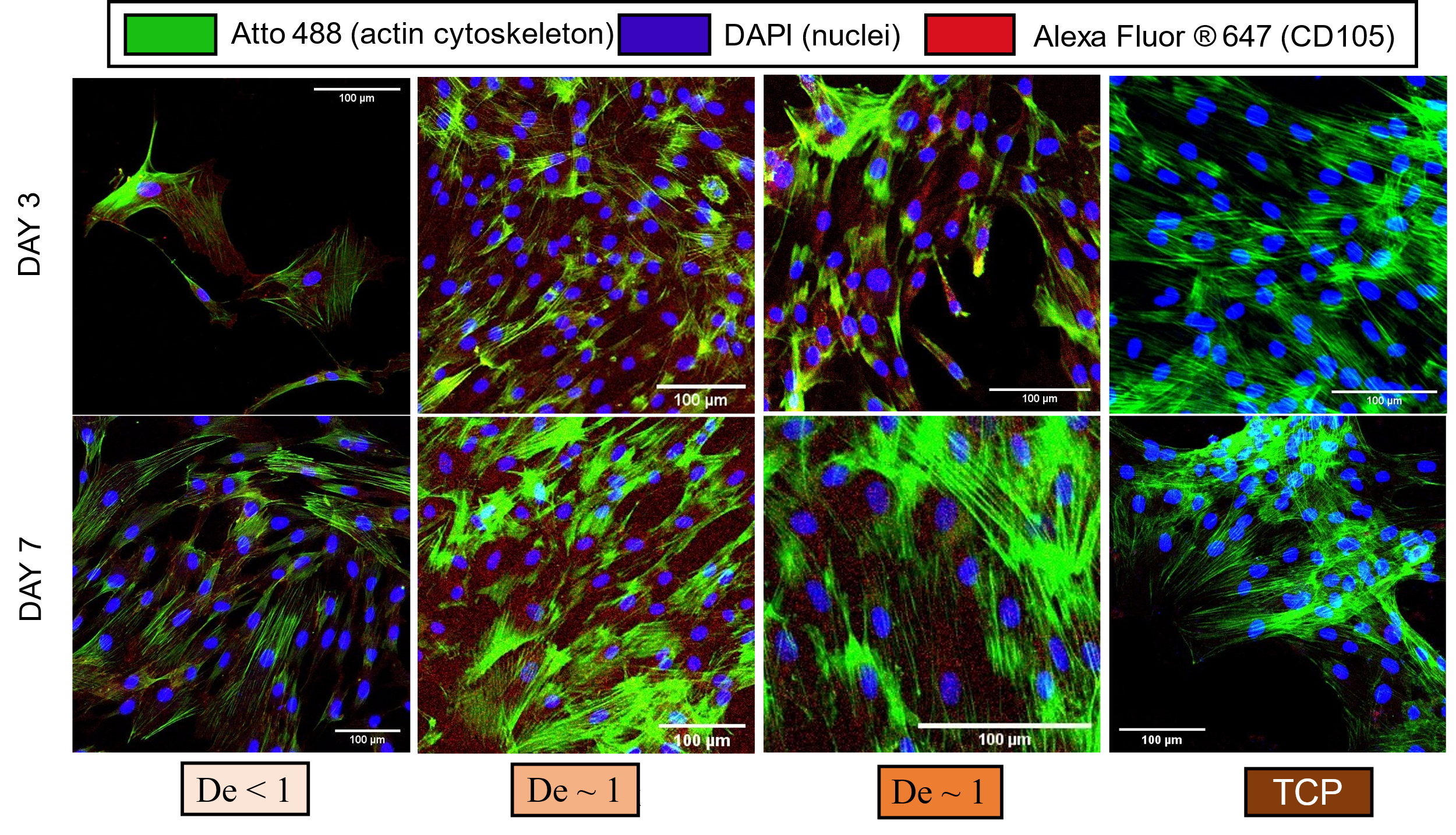
*

Figure S7. CD105 immunostaining: in blue the nuclei, in green the actin cytoskeleton and in red the CD105-associated protein markers.


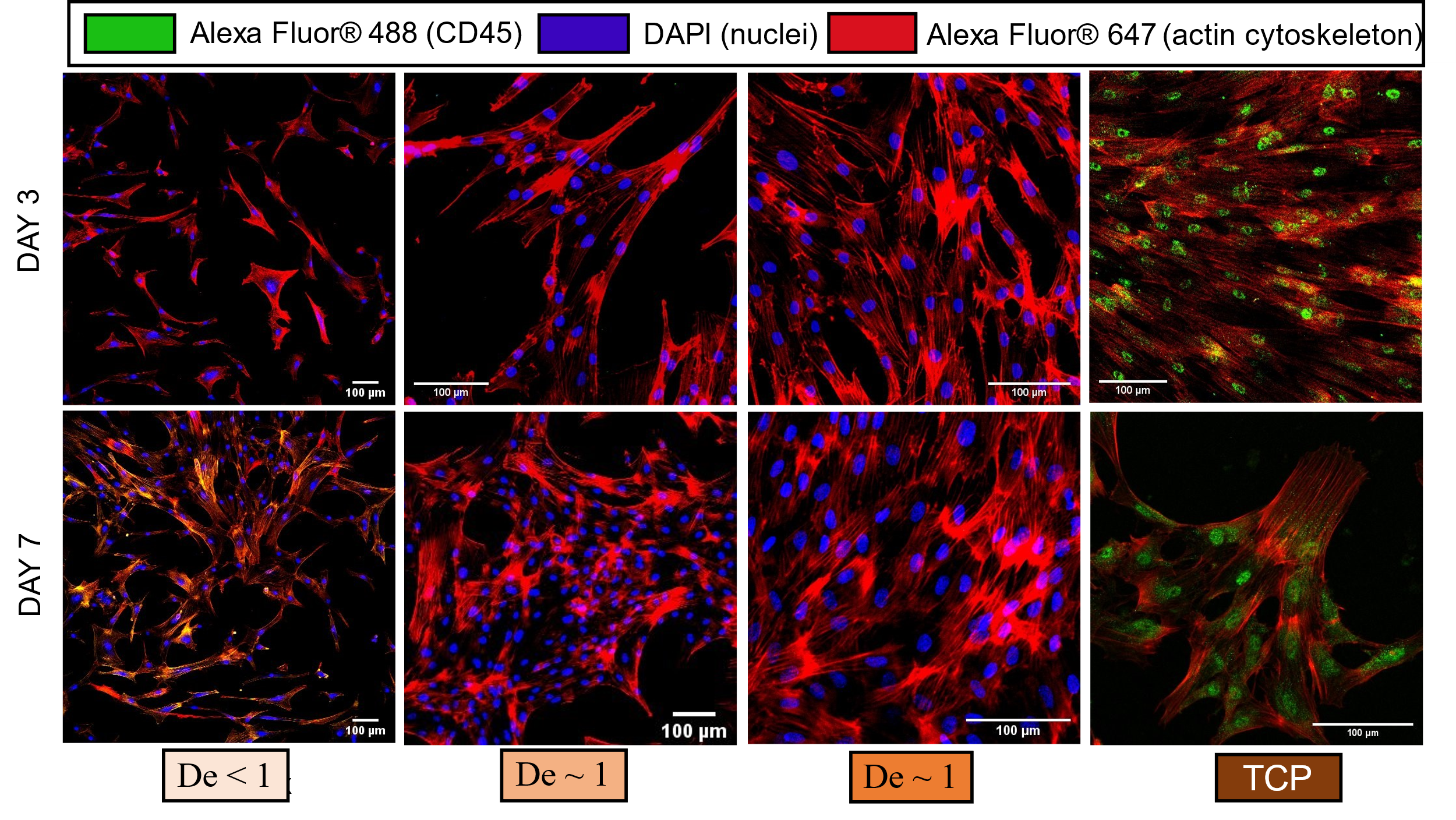


Figure S8. CD45 immunostaining: in blue the nuclei, in red the actin cytoskeleton and in green the CD45-associated protein markers.


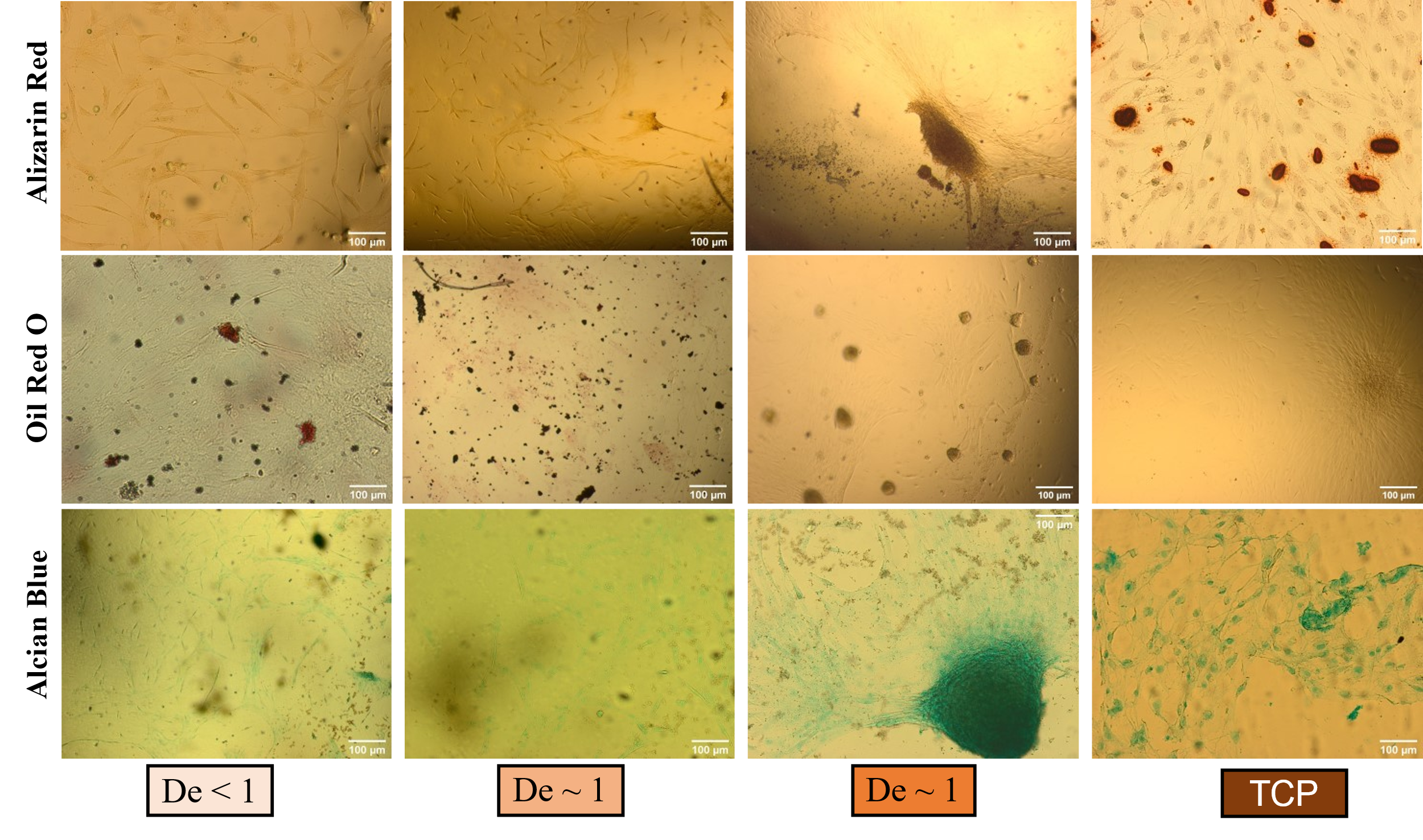


Figure S9. Histological brightfield images after seven days of culture (10X magnification) for detecting calcium deposits (Alizarin Red), lipid droplets (Oil Red O) and GAG agglomerates (Alcian Blue). Calcium deposits are clearly visible in TCP controls, while lipidic droplets can be detected in agarose hydrogels with De<1 (40 mg/mL dextran).

**References**

1. Cacopardo, L., Guazzelli, N. & Ahluwalia, A. Characterizing and Engineering Biomimetic Materials for Viscoelastic Mechanotransduction Studies. *Tissue Eng Part B Rev* **28**, 912–925 (2022).

2. Guazzelli, N., Cacopardo, L., Corti, A. & Ahluwalia, A. An integrated in silico–in vitro approach for bioprinting core–shell bioarchitectures. *Int J Bioprint* **9**, (2023).

3. Cacopardo, L., Guazzelli, N., Nossa, R., Mattei, G. & Ahluwalia, A. Engineering hydrogel viscoelasticity. *J Mech Behav Biomed Mater* **89**, 162–167 (2019).

4. Ike, E. & Ezike, S. C. *ESTIMATION OF VISCOSITY ARRHENIUS PRE-EXPONENTIAL FACTOR AND ACTIVATION ENERGY OF SOME ORGANIC LIQUIDS*. *International Journal of Recent Research in Physics and Chemical Sciences (IJRRPCS)* vol. 5 www.paperpublications.org (2018).

5. Drobny, G., Reid, P. & Engel, T. *Physical Chemistry for the Lide Science*. vol. 26 (Upper Saddle River, NJ: Prentice Hall, 2008).

6. Hagman, J., Lorén, N. & Hermansson, A. M. Effect of gelatin gelation kinetics on probe diffusion determined by FRAP and rheology. *Biomacromolecules* **11**, 3359–3366 (2010).

7. Choi, J. H. *et al.* Evaluation of Hyaluronic Acid/Agarose Hydrogel for Cartilage Tissue Engineering Biomaterial. *Macromol Res* **28**, 979–985 (2020).

8. Pluen, A., Netti, P. A., Jain, R. K. & Berk, D. A. Diffusion of macromolecules in agarose gels: Comparison of linear and globular configurations. *Biophys J* **77**, 542–552 (1999).

9. Cacopardo, L., Guazzelli, N., Nossa, R., Mattei, G. & Ahluwalia, A. Engineering hydrogel viscoelasticity. *J Mech Behav Biomed Mater* **89**, 162–167 (2019).
